# Reply to Abenavoli et al. Comment on “Clayton-Chubb et al. Understanding NAFLD: From Case Identification to Interventions, Outcomes, and Future Perspectives. *Nutrients* 2023, *15*, 687”

**DOI:** 10.3390/nu15132908

**Published:** 2023-06-27

**Authors:** Daniel Clayton-Chubb, William Kemp, Ammar Majeed, John S. Lubel, Alex Hodge, Stuart K. Roberts

**Affiliations:** 1 Department of Gastroenterology, The Alfred Hospital, Melbourne, VIC 3004, Australia; d.clayton-chubb@alfred.org.au (D.C.-C.);; 2 Central Clinical School, Monash University, Melbourne, VIC 3004, Australia; 3 Department of Gastroenterology, Eastern Health, Box Hill, VIC 3128, Australia; 4 Eastern Health Clinical School, Monash University, Box Hill, VIC 3128, Australia

Thank you for your interesting comment [1] on our recent article [2]. The use of phosphatidylcholine as a potential nutraceutical therapeutic in NAFLD makes theoretical sense—choline deficiency is associated with hepatic steatogenesis [3,4], and phosphatidylcholine is an important component of cell membranes with relative deficiency also associated with hepatic steatogenesis [4]. 

While a discussion of all currently studied nutraceuticals was beyond the scope of our article, we agree that the evaluation of the potential therapeutic role of phosphatidylcholine in NAFLD over a longer term has some merit. However, we would also counsel to proceed with caution and to carefully monitor the safety of its administration given that some studies on dietary intake have linked phosphatidylcholine intake to increased all-cause and cardiovascular mortality [5]—which is of particular concern in the NAFLD population. 

Thank you again for your interest in our article. 
